# Efficacy of mindfulness added to treatment as usual in patients with chronic migraine and medication overuse headache: a phase-III single-blind randomized-controlled trial (the MIND-CM study)

**DOI:** 10.1186/s10194-023-01630-0

**Published:** 2023-07-14

**Authors:** Licia Grazzi, Domenico D’Amico, Erika Guastafierro, Greta Demichelis, Alessandra Erbetta, Davide Fedeli, Anna Nigri, Emilio Ciusani, Corso Barbara, Alberto Raggi

**Affiliations:** 1 Neuroalgology Unit and Headache Center, Fondazione IRCCS Istituto Neurologico Carlo Besta, Milano, Italy; 2 Neurology, Public Health and Disability Unit, Fondazione IRCCS Istituto Neurologico Carlo Besta, Via Celoria 11, 20133 Milano, Italy; 3 Department of Neuroradiology, Fondazione IRCCS Istituto Neurologico Carlo Besta, Milano, Italy; 4 Department of Diagnostic and Technology, Fondazione IRCCS Istituto Neurologico Carlo Besta, Milano, Italy; 5 Neuroscience Institute, Consiglio Nazionale delle Ricerche, Padova, Italy

**Keywords:** Chronic Migraine, Medication Overuse Headache, Mindfulness, Quality of Life, Disability, Migraine Prophylaxis, Cost of Illness

## Abstract

**Background:**

Mindfulness gained considerable attention for migraine management, but RCTs are lacking. We aimed to assess the efficacy of a six-sessions mindfulness-based treatment added to treatment as usual (TaU) in patients with Chronic Migraine (CM) and Medication Overuse Headache (MOH) on headache frequency, medication intake, quality of life, disability, depression and anxiety, cutaneous allodynia, awareness of inner states, work-related difficulties, and disease cost.

**Methods:**

In this Phase-III single-blind RCT carried out in a specialty Italian headache center, 177 patients with CM and MOH were randomized 1:1 to either TaU (withdrawal from overused drugs, education on proper medication use and lifestyle issues, and tailored prophylaxis) or mindfulness-based intervention added to TaU (TaU + MIND). The mindfulness-based intervention consisted of six group session of mindfulness practice and 7–10 min daily self-practice. The primary endpoint was the achievement of ≥ 50% headache frequency reduction at 12 months compared to baseline, and was analyzed on an intention-to-treat principle using Pearson’s Chi-Squared test. Secondary endpoints included medication intake, quality of life (QoL), disability, depression and anxiety, cutaneous allodynia, awareness of inner states, work-related difficulties, and disease cost. The secondary endpoints were analyzed using per-protocol linear mixed models.

**Results:**

Out of the 177 participants 89 were randomized to TaU and 88 to TaU + MIND. Patients in the TaU + MIND group outperformed those in TaU for the primary endpoint (78.4% vs. 48.3%; *p* < 0.0001), and showed superior improvement in headache frequency, QoL and disability, headache impact, loss of productive time, medication intake, and in total, indirect and direct healthcare costs.

**Conclusions:**

A mindfulness-based treatment composed of six-week session and 7–10 min daily self-practice added on to TaU is superior to TaU alone for the treatment of patients with CM and MOH.

**Trial registration:**

MIND-CM was registered on clinicaltrials.gov (NCT03671681) on14/09/2018.

**Supplementary Information:**

The online version contains supplementary material available at 10.1186/s10194-023-01630-0.

## Introduction

Chronic Migraine (CM) associated to Medication Overuse Headache (MOH) is characterized by 15 or more days with headache per month and by the overuse of acute medications for at least three months [1]. Patients with CM and MOH experience relevant pain, limitations in daily activities, personal and societal disease burden [2]. Both pharmacological and non-pharmacological treatments are available [3, 4]. Among these, mindfulness-based approaches received attention, and some recent reviews concluded that they may be beneficial, with effects comparable to pharmacological treatments [5, 6]. Mindfulness treatments are aimed to master patients’ ability to intentionally paying attention to the present moment, in a non-judgmental way, so to enable cultivating a full presence in the experience of the moment [6].

Few randomized-controlled trials (RCTs) have been carried out to test the efficacy of mindfulness-based approaches. Wells and colleagues followed-up for 36 weeks 89 patients with migraine (baseline frequency: 4–20 days/month) who were randomized to either education or mindfulness-based stress reduction (MBSR) therapy [7]. The two groups performed similarly for migraine and headache days/month changes, and patients assigned to MBSR showed superior improvements in secondary outcomes such as disability or quality of life (QoL). In a preliminary report from the present study, the MIND-CM study, we presented the feasibility and short-term efficacy of the proposed mindfulness-based protocol as an add-on to treatment as usual (TaU). The results revealed similar between-group drop-out rates, and greater improvements in headache frequency and non-steroidal anti-inflammatory drugs (NSAIDs) intake among patients randomized to receiving mindfulness session added to TaU (TaU + MIND) compared to TaU [8].

RCTs are needed to ascertain the efficacy of mindfulness over other approaches. The herein-presented RCT is grounded on a pilot non-randomized study, in which patients receiving six mindfulness-based sessions only (without pharmacological prophylaxis) reported an improvement in headache frequency and medication intake over 12 months which was similar to that observed in the group of patients who received pharmacological prophylaxis [9]. Thus, we hypothesized and aimed to test that a combined treatment (i.e. TaU + MIND) would be superior to TaU alone in patients with CM and MOH on headache frequency reduction (primary endpoint), and on a set of secondary endpoints, namely: medication intake, QoL, disability and impact, depression and anxiety, cutaneous allodynia, awareness of inner states, work-related activities, and disease cost.

## Methods

### Study design, setting and participants

MIND-CM study was a phase-III single-blind RCT, in which patients with CM and MOH attending our specialty headache center for a structured withdrawal treatment [10], either in ward or in day-hospital, were included. The study was approved by the Ethical Committee of the Besta Institute (approval no. 51/2018), and was registered on clinicaltrials.gov (NCT03671681). The study was launched on November 2018; the last patient was enrolled on December 2021; the last follow-up was completed on November 2022.

The inclusion criterion was the diagnosis of both CM and MOH (codes 1.3 and 8.2 of the International Classification of Headache Disorders, 3rd edition [1]) with a close temporal relation. Therefore, in the previous three months, they had 15 or more headache days per month, of whom at least 8 with migraine-like features, and overused one or more class of symptomatic medications. Exclusion criteria were: psychiatric comorbidities of psychotic area; pregnancy; secondary headaches; withdrawal from MOH twice or more in the previous two years; any previous experience with mindfulness.

### Sample size calculation

The sample size was determined on the basis of previous experience of the team, hypothesizing that 48% of patients allocated to TaU would achieve ≥ 50% headache-day reduction after 12 months, and that adding mindfulness might increase this figure by 20%. We set alpha at 0.05 and power at 80%, and determined that 75 patients per group were needed; considering that up to 12% of patients might be lost at follow-up, we determined that 170 patients should be randomized. The G-Power software was used for sample size calculation.

### Randomization

Patients were randomized 1:1 to either TaU or TaU + MIND using a computer-generated list of random numbers. The study neurologist who enrolled the patients and followed them (D.D.) was not involved in the randomization procedure and remained blind to allocation. A single researcher (A.R.) prepared the randomization list and a set of opaque envelopes two months before the study beginning. Mindfulness sessions were administered by the second study neurologist (L.G., a leading expert in mindfulness and other behavioral treatment for headache). A.R. and the other researchers randomized patients to the two groups and handled data collection.

After the screening visit, on the second day of withdrawal, patients meeting the selection criteria were asked to voluntarily join the study. Those who accepted signed an informed consent form and were randomized. The consent for fully explained the study purpose and the fact that the evaluating neurologist was blind to patients’ allocation. Therefore, at each follow-up, data referred to primary and secondary endpoints were collected by one of the researchers, and patients were reminded not to disclose their allocation to TaU or TaU + MIND to the study neurologist who performed the clinical evaluations (D.D.).

### Intervention: TaU condition

TaU consisted of three main elements: overused drugs withdrawal; education on proper medication use, and prescription of tailored prophylaxis.

Withdrawal was carried out in a hospital setting, either in day-hospital or ward, and lasted 5–8 days. It consisted of: abrupt withdrawal of overused symptomatic medications; intravenous hydration for the whole period; intravenous steroids and ademetionine for 5 days followed by oral prescription of steroids for other 3–5 days; oral benzodiazepines; intravenous methoclopramide or indomethacin if needed for intense rebound headache.

Patients’ education was focused on both proper medication use and lifestyle issues. Patients were encouraged to use acute medications only for headaches that were very disabling and painful, operationally defined as 8 or more on a 0–10 pain scale (no pain at all – pain as bad as it can be). As the first-line treatment, Eletriptan (40 mg) and/or Almotriptan (12.5 mg) were recommended, and Indomethacin (50 mg) as the second line; other NSAIDs should be taken if they had already proven to be effective. Finally, in any case, they were strongly advised to avoid opioids. With reference to lifestyle issues, patients were encouraged to engage in a moderate level of aerobic physical activity for approximately 45 min twice per week, to maintain a suitable level of hydration, to consume 3 meals each day on a regular basis (emphasizing breakfast) and, finally, to care for sleep hygiene, i.e. sleeping around 7–8 h per night and trying to be consistent with timetables for sleep time.

By the end of withdrawal treatment, patients were prescribed tailored prophylaxis based on their clinical characteristics and previous experiences with single compounds. The study used marketed pharmacological compounds that are commonly prescribed in our center on a daily basis (e.g. antiepileptic, antidepressant or anti-hypertensive drugs, OnabotulinumtoxinA, and Calcitonin gene-related peptide monoclonal antibodies – see Burch 2021 for a review [11]), and possibly on nutraceuticals, such as magnesium pidolate, Q10 coenzyme, or B2 vitamin.

### Intervention: mindfulness-based sessions

The sessions with patients involved mindfulness practice, and patients were trained to meditate; moreover, notions and information on acceptance of their condition and invitations to be more flexible were presented through examples and metaphors. The program consisted of 6 weekly sessions, 90 min each. Small groups of 6–8 patients were invited to sit in a quiet room. The sessions were guided by an experienced mindfulness instructor.

In the first session, patients were encouraged to introduce themselves, and share information about their migraine and disease history. They were also instructed to find the right posture for starting the practice by adjusting the chair and the position of their back, legs, and feet on the floor. The Mindfulness practice was introduced in the first session: patients were invited to close their eyes and take 2–3 deep breaths. After this, they were invited to let their breath adjust to the best rhythm for that moment. The first practice session lasted 5 min, leaving patients time enough to explain their sensations and impressions. Each practice session ended with a discussion of any unpleasant sensations, if needed.

From the second session onwards, a specific focus was given to mindfulness practice, with an emphasis on its value and the definition: the significance of the practice for facing pain and medication use, the importance of being more flexible, and the importance being trained in mindfulness as part of the treatment strategies for migraine. Such a practice was intended to enhance patients’ ability to recognize their internal feelings and accept them in a non-judgmental way. Acquiring such an ability should enable patients to recognize when they need headache medications and when they do not, and it thus addresses the pain-pill automatism. The mindfulness sessions increased in duration: from 5 min in the first ones, they reached 25 min towards the end of the intervention.

Across the sessions, patients were trained to focus on breathing and to reduce judgments by acknowledging arising thoughts, feelings, and emotions, without any emotional reaction. Patients were instructed to gently refocus on their breathing if their attention disengaged, and trained not to change their breathing rate voluntarily.

Patients were invited to notice any atypical or unpleasant feeling during the sessions and also during home practice. At the beginning of every session, patients were asked about the previous week, the symptoms they might have experienced – including both migraine-related ones and some psychological reactions they might have noticed, e.g., feelings of anxiety – and how was the therapy going on. As the practice progressed, patients were invited to try practicing at home by using a few-minute session that focused just on breathing from the third session onwards. The home practice session began with a 3-minutes duration and reached the target of 7–10 min within a few days.

On the fifth and sixth sessions, the acquired knowledge was integrated and further connected to the recognition of internal sensations as they relate to headache pain and to identifying situations in which patients need to rely on drugs for managing their headaches or when they do not. In the last two sessions, the mindfulness meditations lasted 25 min. Patients were provided with a 12-minute audio file to use at any time after the structured intervention concluded. This allowed them to practice for 7–10 min, or even a little longer over the course of the 12 months’ follow-up.

### Follow-up assessments

Follow-up assessments, at 3, 6 and 12 months from enrollment, were conducted in person whenever possible. During the critical phases of the COVID-19 pandemic, tele-visits were implemented: patients were contacted by phone, and received modifiable files with the protocol questionnaires.

### The research protocol

Headache frequency was derived from structured headache diaries. The primary endpoint was the achievement of 50% or more reduction in headache frequency at 12 months compared to baseline.

Secondary endpoints included variations in the following: headache frequency (change in number of days and percentage change); QoL, assessed by the Migraine-Specific Quality of Life Questionnaire, version 2.1 (MSQ v2.1) [12, 13]; disability, assessed by the Migraine Disability Assessment (MIDAS) [14, 15], and by the 12-item WHO Disability Assessment Schedule (WHODAS-12) [16]; headache impact, assessed by the six-item Headache Impact Test (HIT-6) [17]; symptoms of depression and anxiety, assessed by the Beck Depression Inventory-II (BDI-II) and the State-Trait Anxiety Inventory, respectively (STAI-Y) [18–21]; cutaneous allodynia, assessed by the 12-items Allodynia Symptoms Checklist (ASC-12) [22]; self-awareness, assessed by the Mindful Attention and Awareness Scale (MAAS) [23]; impact on work-related activities, calculated by the HEADWORK questionnaire [24], and by a day-equivalent loss of productive time (LPT) measure which accounts for both lost workdays and days worked with headache, as developed in a protocol dedicated to MOH cost calculation [25]; symptomatic medications intake, herein detailed in terms of NSAIDs, triptans, and total drug intake; disease cost, including indirect, direct healthcare, and direct non-medical costs.

A detailed description of the research protocol is included in Supplementary Methods 1 (see Additional file 1).

### Statistical analyses

#### Descriptive statistics and baseline comparisons

Descriptive statistics of the study sample were obtained at baseline for socio-demographic characteristics (age, sex, marital status, years of education, education level), migraine and CM duration, previous withdrawals, and working setting (context, years working, years working at current place, weekly working hours, company size). Data were tabulated by treatment group and presented as absolute numbers (percentages) for categorical variables and as medians (first and third quartile: Q1-Q3) for quantitative variables. Differences between groups were inspected by Fisher’s exact test for categorical variables, and by Mann-Whitney U test for quantitative data. Normality of data was tested by Shapiro Wilks test.

#### Endpoint analyses

The primary endpoint was analyzed on an intention-to-treat (ITT) principle using Pearson’s Chi-Squared test. On ITT analysis, all patients should be analyzed as part of the group to which they were randomly assigned. To perform this analysis, missing data were imputed using multiple imputations by chained equations. The predictive mean matching imputation method was implemented with 20 sets of imputations.

The secondary endpoints were independently analyzed using a per-protocol analysis approach by means of linear mixed models. Using these models, each secondary endpoint is considered as dependent variable at each time point. They have the advantage to use all available data, thus all patients are included regardless they completed the trial or not. All models had a random intercept for patients (to account for within-person clustering of repeated observations) with unstructured covariance structure, and were adjusted for the following baseline characteristics: age (years), sex, years of education, CM duration, working status, and previous withdrawal. An interaction term between time and treatment group was included to test the differences between groups.

Finally, we calculated whether the mindfulness add-on was associated to higher rates in achieving clinically meaningful improvement at HIT-6 score (i.e. change in score ≥ 6, specific for CM [26]) at the three time points of follow-up using Pearson’s Chi-Squared test.

Analyses were performed with Stata software, version IC 15.1 (StataCorp LLC). For all tests and models significance level was set at *p* = 0.05, two-tailed.

## Results

A total of 177 patients were randomized to the two groups (see Table 1 for baseline description): 89 to receive TaU, and 88 to receive TaU + MIND. At baseline, patients in the TaU group had a median of 60 headache days/three months (Q1-Q3: 50–75), those in the TaU + MIND group had 65 (Q1-Q3: 54–80).

**Table 1:** Descriptive statistics of the study sample by group

		TaU Group (*N* = 89)	TaU + MIND Group (*N* = 88)	Total (*N* = 177)	*p*-value
**Age**	Median (Q1, Q3)	49.5 (40.1, 54.0)	46.4 (40.3, 55.1)	47.9 (40.1, 54.2)	0.435
**Sex**	Males	11 (12.4%)	9 (10.2%)	19 (10.7%)	0.813
	Females	78 (87.6)	79 (89.8%)	158 (89.3%)	
**Marital Status**	Never Married	13 (14.6%)	22 (25.0%)	35 (19.8%)	0.064
	Married/Cohabitating	66 (74.2%)	50 (56.8%)	116 (65.5%)	
	Separated/Divorced	7 (7.9%)	14 (15.9%)	21 (11.9%)	
	Widowed	3 (3.4%)	2 (2.3%)	5 (2.8%)	
**Years of formal Education**	Median (Q1, Q3)	13.0 (13.0, 17.0)	13.0 (13.0, 17.0)	13.0 (13.0, 17.0)	0.316
**Education Level**	Primary School	1 (1.1%)	0 (0.0%)	1 (0.6%)	0.753
	Secondary School	20 (22.5%)	17 (19.3%)	37 (20.9%)	
	High School	43 (48.3%)	40 (45.5%)	83 (46.9%)	
	Any College	20 (22.5%)	23 (26.1%)	42 (24.3%)	
	Post-Lauream degree	5 (5.6%)	8 (9.1%)	13 (7.3%)	
**Employment status**	Employee	47 (52.8%)	48 (54.5%)	95 (53.7%)	0.967
	Employer/Manager	6 (6.7%)	6 (6.8%)	12 (6.8%)	
	Private practitioner	7 (7.9%)	9 (10.2%)	16 (9.0%)	
	Other (incl.student)	9 (10.1%)	7 (8.0%)	16 (9.0%)	
	Not employed	20 (22.5%)	18 (20.5%)	38 (21.5%)	
**Years of Work**	Median (Q1, Q3)	24.0 (17.0, 30.0)	23.5 (15.0, 31.0)	24.0 (15.0, 30.0)	0.833
**Weekly Worked Hours**	Median (Q1, Q3)	40.0 (36.0, 48.0)	40.0 (30.0, 43.0)	40.0 (34.0, 45.0)	0.113
**Company Size**	Micro	20 (29.0%)	23 (32.9%)	43 (30.9%)	0.800
	Small	8 (11.6%)	11 (15.7%)	19 (13.7%)	
	Medium	13 (18.8%)	11 (15.7%)	24 (17.3%)	
	Large	28 (40.6%)	25 (35.7%)	53 (38.1%)	
**Migraine Duration (years)**	Median (Q1, Q3)	28.0 (20.3, 39.5)	28.6 (16.2, 35.7)	28.5 (18.6, 37.4)	0.238
**CM Duration (years)**	Median (Q1, Q3)	14.9 (4.5, 24.4)	14.0 (5.6, 20.7)	14.6 (4.9, 22.2)	0.651
**Headache days/last three months**	Median (Q1, Q3)	60.0 (50.0, 75.0)	65.0 (54.0, 80.0)	60.0 (50.0, 80.0)	0.250
**Previous Withdrawals**	No. of patients (%)	21 (23.6%)	30 (34.1%)	51 (28.8%)	0.138
**No. of Previous Withdrawal**	1	12 (57.2%)	16 (53.4%)	28 (54.9%)	0.570
	2	2 (9.5%)	7 (23.3%)	9 (17.6%)	
	3	5 (23.8%)	5 (16.7%)	10 (19.6%)	
	4	2 (9.5%)	1 (3.3%)	3 (5.9%)	
	7	0 (0.0%)	1 (3.3%)	1 (2.0%)	

*Notes.* Mann-Whitney U test for quantitative data; Fisher’s exact test for categorical variables. Company size: Micro 1–9, Small 10–49, Medium 50–249, and Large 250 + employees

**Fig. 1:**
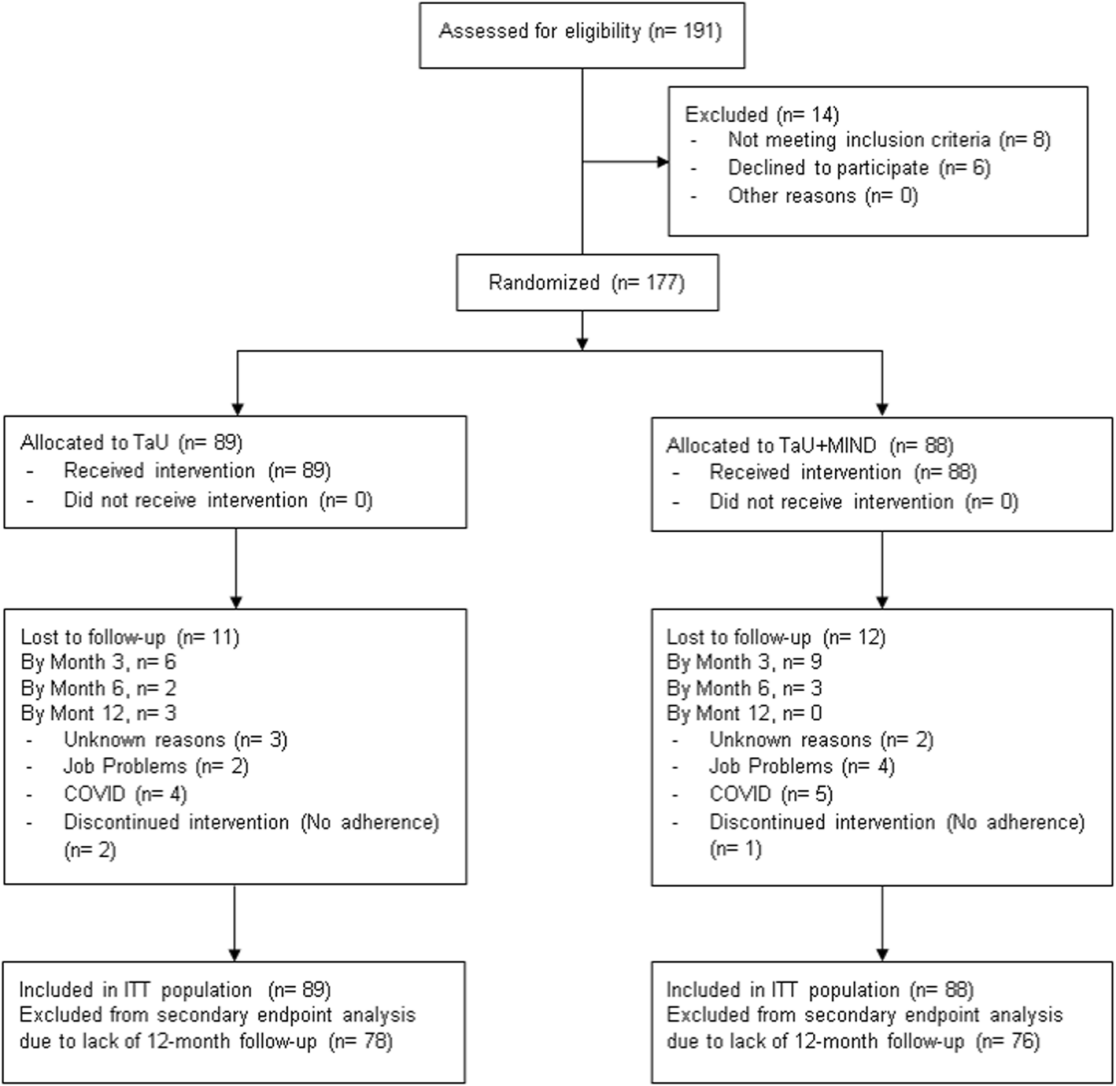
MIND-CM study flowchart

Figure 1 shows the study flowchart. Twenty-three patients dropped-out: 11 from the TaU group (12.4%), of whom six by month 3; 12 from the TaU + MIND group (13.6%), of whom nine by month 3. No adverse events were reported by the patients.

### Primary endpoint analysis

In the ITT analysis, the primary endpoint was achieved by 43/89 patients (48.3%) in the TaU group and by 69/88 patients (78.4%) in the TaU + MIND group (Chi-Squared = 17.2; *p* < 0.0001).

### Secondary endpoints analyses

**Fig. 2:**
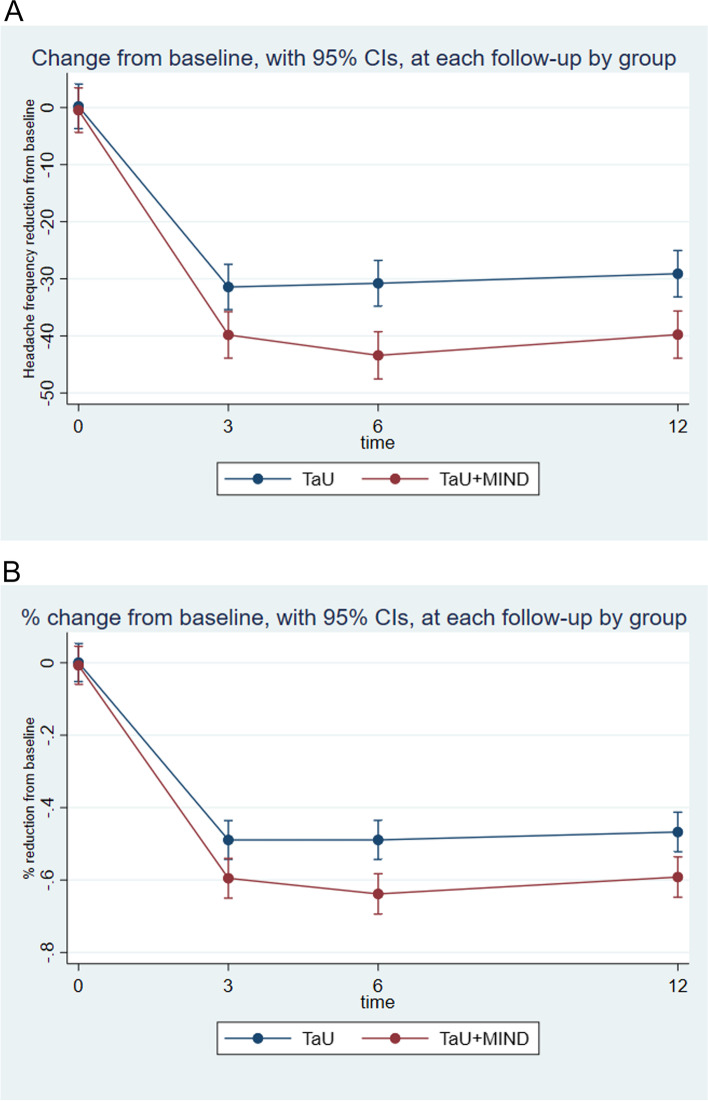
Time by group analysis for change in headache frequency and percentage change from baseline to month 12. *Notes.* **A**, Headache days’ change from baseline to months 3, 6 and 12; **B**, Headache days’ percentage change from baseline to months 3, 6 and 12

Figure 2 shows headache frequency reduction and percentage reduction over 12 months: patients in the TaU + MIND group outperformed those in the TaU group (*p* < 0.0001 and *p* = 0.0001, respectively).

Patients in the TaU + MIND group outperformed those in the TaU group at MSQ and MIDAS scores at the 12-month follow-up only, whereas the two groups overlapped throughout the study at WHODAS-12 and HIT-6 scores (see Supplementary Figs. 1–4 for MSQ, MIDAS, WHODAS-12 and HIT-6 time by group interactions in Additional file 2). Patients in the TaU + MIND group achieved significantly better results than those in TaU for the achievement of HIT-6 clinically meaningful reduction at each follow-up (see Supplementary Table 1 in Additional file 2). No time by group interaction was found at BDI-II, STAI-Y, ASC-12, MAAS, and HEADWORK questionnaires (see Supplementary Figs. 5–9 in Additional file 2).

**Fig. 3:**
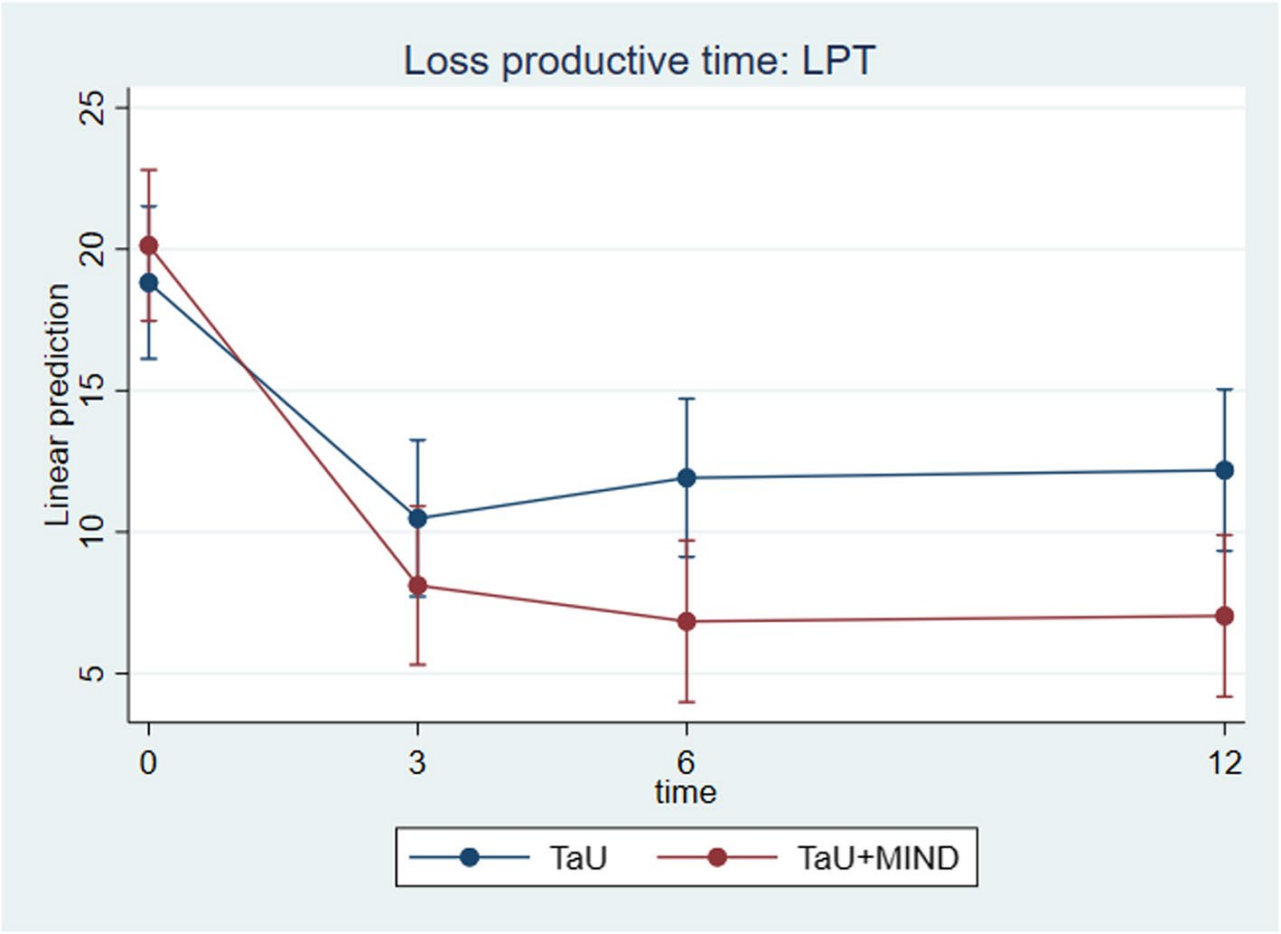
Time by group analysis for day-equivalent loss of productive time (LPT) from baseline to month 12

Patients in the TaU + MIND group showed significantly lower total disease, indirect and direct healthcare cost than those in the TaU group (*p* < 0.0001, *p* = 0.0004 and *p* = 0.0071, respectively; see Supplementary Fig. 10 in Additional file 2). Also, patients allocated to TaU + MIND group outperformed those in the TaU group for LPT (Fig. 3; *p* = 0.0086).

**Fig. 4:**
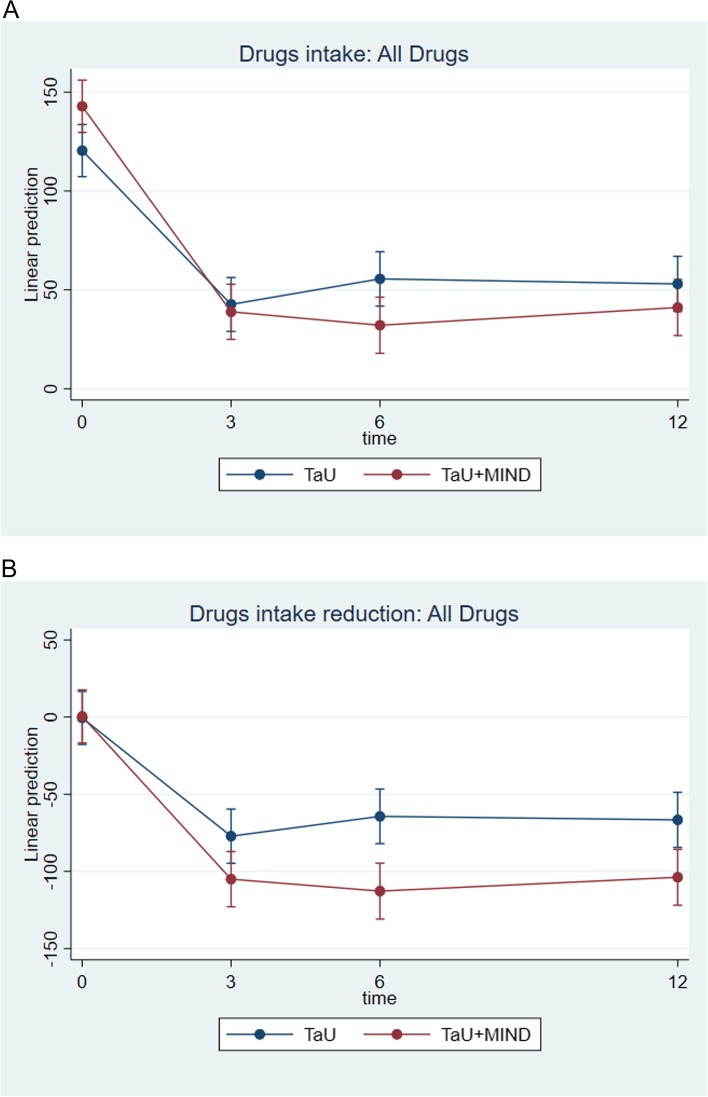
Time by group analysis for all-drugs intake from baseline to month 12. *Notes.* **A**, All-drugs intake between baseline and month 12; **B**, Decrease in all-drugs intake from baseline to month 3, 6 and 12

No time by group effect was found for drug intake. However, patients allocated to TaU + MIND outperformed those in the TaU group for the reduction in total drug intake (*p* = 0.0001) and in NSAIDs intake (*p* < 0.0001) from baseline. Figure 4 shows the total drugs intake and the corresponding reduction from baseline; Supplementary Figs. 11–12 show intake and intake reduction from baseline for NSAIDs and triptans (see Additional file 2).

## Discussion

The results of this phase-III single-blind RCT, demonstrated that a mindfulness-based protocol added-on to TaU for the treatment of CM associated to MOH produced a superior and statistically significant reduction in headache frequency, total drug intake and NSAIDs intake compared to baseline compared to TaU alone. Patients allocated to the TaU + MIND group also achieved more frequently a meaningful reduction in headache impact, as defined by a reduction ≥ 6 points in HIT-6 score, and outperformed those in the TaU group with regard to LPT, indirect, direct healthcare, and total costs throughout the study. Finally, they showed better QoL and disability scores (as measured by the MIDAS) at 12 months only.

Mindfulness gained considerable attention for migraine management and some reviews have been carried out [3, 5, 6, 27–29]. Results are partly contrasting, but they mostly showed that mindfulness produced a significant improvement in headache frequency (with two exceptions [27, 29]), pain intensity, medication intake, disability, quality of life, depression and anxiety, pain catastrophizing and fear of pain, and self-efficacy. However, the presence of many small pilots and few RCTs, with small samples, mixed patient populations and outcomes constitute a relevant bias: thus a call for RCTs with adequate samples and well-defined outcomes has often been made.

The MIND-CM study is, to date, one of the largest on behavioral treatments, and the largest on mindfulness in patients with migrainous headaches. Patients allocated in the TaU + MIND group achieved more frequently, than those receiving TaU only, 50% or more headache frequency reduction (78.4% vs. 48.3%): such a result was superior to our hypotheses, which accounted for a 20% higher rate, and is corroborated by the time by group analyses on headache impact, LPT, medications intake, and cost.

A delta of approximately 3.5 days/month between the two groups was found on headache frequency, being the reduction by around 30 days for patients in TaU and 40 in those in TaU + MIND. The mean headache days’ percentage reduction was comprised between 59% and 64% among patients in the TaU + MIND group. This result is wider compared to that of previous investigations, which often found either no [7, 9, 30] or lower differences when mindfulness alone was compared to other treatments: Seminowicz reported a decrease from 7.8 to 4.6 headache days/month at 20 weeks, i.e. 40% [31]; Simshäuser reported a decrease from 8.2 to 6.0 headache days/month at 7 months, i.e. 30% [32]. The reduction we found is broader likely because we added mindfulness to TaU and, in fact, patients randomized to the TaU + MIND group achieved a 20–23% higher headache frequency reduction compared to those in TaU.

The clinical improvement was corroborated by some improvement in disability (as measured by the MIDAS) and in QoL at 12 months only, along with a higher rate of achievement of clinical meaningful HIT-6 score reduction, and by a superior overall LPT improvement. Improvements in disability and QoL were not systematically retrieved in previous studies contrasting mindfulness treatment participants to controls [7, 31, 33–35], and have to be positively interpreted despite patients receiving TaU + MIND still experienced a relevant impact of their condition at 12 months (i.e. MIDAS and HIT-6 scores above 21 and 55, respectively).

Of specific interest is the superior outcome of patients in the TaU + MIND group on LPT, reduced intake of symptomatic medications and disease cost, all of which have not been systematically investigated before. Our mindfulness-based protocol produced a sizeable LPT reduction, which moved from 20 to 7 days-equivalent per trimester (the average reduction being 60–66%), which is similar to what was observed on headache frequency, and superior to that of patients allocated to TaU. The greater improvement in LPT is clearly connected to the superior improvement in indirect cost, which represents the largest part of migraine-associated costs [25, 36, 37]. In the TaU + MIND group, indirect costs dropped from 1890€ to 400€, vs. 1670€ to 1120€ in the TaU group.

Additionally, patients in the TaU + MIND group outperformed those in TaU for medication intake, and specifically NSAIDs, reduction. This is of relevance, considering medication overuse role in migraine chronification [38, 39], and that tackling the pain-pill automatism is a core content of our mindfulness protocol: the effect is evident as those in the TaU + MIND group reduced the amount of intakes per headache day by approximately 23% (from 2.26 to 1.73), whereas those in TaU by 6% (from 1.87 to 1.75). Despite we did not find any relevant difference at MAAS in the time by group analysis, a within-subjects comparison showed an improvement in patients’ level of awareness of mental states over the 12-months period, which might explain the greater improvement in medication intake. The fact that NSAIDs, and not triptans, intake was significantly more reduced in patients allocated to TaU + MIND is thought to be associated to an enhanced patients’ ability to recognize when medications are really needed.

Considering the unpredictable course of migraine headaches, and the difficulties connected to their occurrence, addressing patients’ perception of these difficulties and increasing their ability to handle them is of core relevance. We hypothesize that this enhanced ability led to the reduction in medication intake and to an improved patients’ ability to work, both of which were linked to the superior reduction in disease costs, whose difference was 690€ at 6 months and 850€ at 12 months. Despite a cost-efficacy analysis is out of the scope of this work, it seems there is room enough to compensate for the cost of a regular implementation of the mindfulness protocol herein proposed.

Some limitations need to be considered. First, the study was based in a single center with patients with very severe CM and MOH who attended a structured withdrawal at a specialty headache center: this partially limits the generalizability of our results. Second, COVID-19 pandemic partly disrupted our RCT. In fact, most of the drop outs from the TaU + MIND group were registered in the first trimester due to the development of COVID-19 or to the restrictions in mobility and in access to hospitals. Moreover, we had to switch to tele-visits instead of in-person visits. Third, we did not systematically report adverse events due to mindfulness practice. Behavioral treatments are deemed to be free from side effects [5], and we did not record any relevant event during the sessions, such as panic attacks. Fourth, although we implemented, on the occasion of follow-up visits, a question to address adherence to the 7–10 min daily self-practice, we decided not to include it in the analytical plan, for different reasons. These include: the recall bias in relation to the time-point of assessment (i.e. the previous three months for the first two follow-up evaluations, the previous six months for the last one); the impact of COVID-19 pandemics, which disrupted daily habits of patients; the low level of reliability compared to modern approaches, like applications for mobile devices. The use of e-diaries tracking lifestyle factors with a possible impact on headaches, and evaluation of headache patterns showed to positively impact on headache and migraine frequency [40], and might therefore support standard headache care also for tracking the adherence to mindfulness home self-practice. Fifth, a specific comment on the TaU intervention is needed: it is based on a plurality of interventions, which showed to positively impact on headache course and medication intake, with patients showing maintenance of improvement up to three years [10, 41]: however, some variables were left uncontrolled, and specifically prophylaxis and education. With regard to the first, differences in the prescribed prophylaxis, which was based on marketed compounds and was subject to change over the study period according to clinical needs, may exist. With regard to the second, the degree to which education was delivered to single patients might be different in terms of time dedicated, for example in consideration that patients were enrolled both from the ward and from the day-hospital.

## Conclusions

The MIND-CM study showed that a mindfulness-based treatment composed of six-week session and 7–10 min daily self-practice added on to TaU produced greater improvements in several relevant outcomes, namely headache frequency, medication intake, headache impact, LPT, disease cost and a better output in disability and QoL at 12 months from baseline in a large sample of patients with CM and MOH. Mindfulness should therefore be considered as part of the regular treatment of patients with CM and MOH.

## Supplementary Information

**Additional file 1.**

**Additional file 2.**

## Data Availability

The datasets used and analyzed during the current study is available at 10.5281/zenodo.8119179.
