# Supplementary material for: Efficacy of mindfulness added to treatment as usual in patients with chronic migraine and medication overuse headache: a phase-III single-blind randomized-controlled trial (the MIND-CM study)
Source: J Headache Pain. 2023 Jul 14;24(1):86. doi: 10.1186/s10194-023-01630-0 (PMC10347788; doi:10.1186/s10194-023-01630-0)
Supplement: Supplementary file 1 — Additional file 1. [file 10194_2023_1630_MOESM1_ESM.docx]

**Additional File 1**

Online-only Supplementary Material to “Efficacy of Mindfulness added to treatment as usual in patients with Chronic Migraine and Medication Overuse Headache: A Phase-III Single-Blind randomized-controlled trial (the MIND-CM study)”

**Authors**: Licia Grazzi, Domenico D’Amico, Erika Guastafierro, Greta Demichelis, Alessandra Erbetta, Davide Fedeli, Anna Nigri, Emilio Ciusani, Barbara Corso, Alberto Raggi.

**Journal:** Journal of Headache and Pain

**SUPPLEMENTARY METHODS 1.**

**Description of the full research protocol**

The Migraine-Specific Quality of Life Questionnaire, version 2.1 (MSQ v2.1) [1,2] was used to assess quality of life (QoL). It is a 14-item questionnaire designed to measure how migraines affect and/or limit daily functioning across 3 domains: Role-Restriction (RR), composed of 7 items assessing how migraines limit the patient's daily social and work-related activities; Role-Prevention (RP), composed of 4 items assessing how migraines prevent daily activities; Emotional Function (EF), composed of 3 items assessing the emotions associated with migraines). Participants respond to items using a 6-point scale, ranging from “none of the time” to “all of the time” which correspond to scores between 1 and 6. Raw dimension scores are computed as a sum of item responses and are rescaled to a 0-100 scale, where higher scores indicate better QoL.

Disability was assessed with the Migraine Disability Assessment (MIDAS) [3,4], and the 12-item WHO Disability Assessment Schedule (WHODAS-12) [5,6]. The MIDAS is composed of seven questions referred to the previous 3 months, and require patients to refer the number of days in which they experienced the limitations addressed in each of them. The first two items address limitations in paid and schoolwork activities, and patients have to report the number of days in which they were completely unable (question 1) or limited in more than half of their ability (question 2) to carry out paid and schoolwork activities. The third and the fourth items address the same concept, but referred to household work. The fifth item addresses the number of days in which headaches had an impact on leisure activities with family or in social situations. The last two questions investigate the total number of days with migraine attacks and the mean pain intensity. MIDAS score is the sum of responses to questions 1–5 and four severity grades are available: minimal (0–5), mild (6–10), moderate (11–20), and severe (> 21) disability. The WHODAS-12 is a brief version of the WHODAS 2.0, composed of 12 items that address the six main domains of the full questionnaire, namely cognition, mobility, self-care, getting along, life activities, and participation. Responses are rated on a five-point scale (no difficulty–extreme difficulty) based on the judgments of their ability to perform the 12 activities over the past 30 days. A summary index, with scores ranging between 0 and 100, is available; higher scores indicate a higher disability.

The six-item Headache Impact Test (HIT-6) [7] measures the impact of headaches on patients’ ability to function at work, at school, at home, and in social situations. Each of its six items is rated on a 5-point scale, ranging from “never” to “always”. The total scores range between 36 and 78. HIT-6 scores ≤49 indicate little to no impact, scores between 50 and 55 indicate some impact, scores between 56 and 59 indicate substantial impact, and scores ≥60 indicate severe headache impact.

Depression symptoms were measured with the Beck Depression Inventory-II (BDI-II) [8,9], a 21-item inventory addressing both the cognitive (e.g. pessimism, guilt, self-criticism, and self-esteem) and the somatic-affective component (e.g. loss of interest, loss of energy, changes in appetite and sleep, agitation, and crying) of depressed mood. Each item includes four descriptors (0-3 scale) addressing symptoms’ severity, and the total BDI-II score range is 0–63, with higher scores reflecting higher depressive mood. BDI-II total score in the range of 14–19 indicates mild depressive symptoms, 20–28 indicates moderate depressive symptoms, and a score ≥29 indicates severe depressive symptoms.

Anxiety symptoms were measured with the State-Trait Anxiety Inventory Y-form (STAI-Y) [10,11]. It is composed of 40 items that form two subscales, namely state anxiety (i.e. how anxious the person is at the time the questionnaire is administered) and trait anxiety (i.e. how anxious the person is on a regular basis). Each item is rated on a 1–4 scale, and both trait and state scores range from 20 to 80, with higher scores indicating higher levels of anxiety. Raw scores were converted into T-Scores (with mean = 50 and SD = 10) on the basis of age and gender-based normative Italian scores: clinically relevant anxiety was defined as a T-Score >60.

The 12-items Allodynia Symptoms Checklist (ASC-12) [12] is composed of 12 items referring to the presence of cutaneous allodynia (i.e., the extent to which cutaneous stimulation that ordinarily would not produce pain, such as wearing eyeglasses, is experienced as pain in the course of a headache). Respondents are asked to rate how often they experience heightened pain or unpleasant skin sensations during each of them when having a severe headache. Each item is rated on a 0-2 points scale, where 0 is used for either “does not apply to me” and for “never/rarely”, 1 is used for “less than half of the time”, and 2 for “half of the time or more”. Score values range from 0 to 24, with 0–2 indicating no symptoms of allodynia, 3–5 indicating mild symptoms, 6–8 reflecting moderate symptoms, and scores ≥9 indicating severe allodynia.

The Mindful Attention and Awareness Scale (MAAS) [13] is designed to assess core aspects of mindfulness, i.e. receptivity to focusing on, being aware of, and observing what is occurring in the present. It is composed of 15 items, each rated on a 6-point scale, rated between 1 (almost always) and 6 (almost never). Total scores range between 15 and 90, with higher scores reflecting higher levels of mindfulness.

The impact on work-related activities was measured with the HEADWORK questionnaire [14], a 17-item, two-scale questionnaire. In the first HEADWORK scale, titled “Work-related difficulties”, 11 items address the degree to which headaches affect different activities. Each item is rated on a 1-5 scale, ranging from “no difficulties” to “extreme difficulty/cannot do”, and examples include using the computer or talking and interacting with other people, and general skills, such as problem solving. The second scale is named “Factors contributing to work difficulties” and is composed of 6 items that address the environmental conditions of the workplace and colleagues’ attitudes. Items are rated on a 1-5 scale ranging from “None” to “Completely”. The first scale score range is 11-55 and the second is 6-30, but both were rescaled on a 0-100 range.

The loss of productive time (LPT) was calculated as “day-equivalent”. Patients were asked to indicate the number of missed workdays (absenteeism) and reduced productivity days (presenteeism) over the previous three months. For the latter, they were also requested to estimate their overall performance level on a 1-99% scale: the percentage needed to get to 100% was used as a coefficient to calculate the loss of productivity in days worked with headache. For example, if a patient missed 5 days and worked 20 days with 60% productivity, then LPT would be 5+(20*0.4), i.e. 13 days-equivalent.

We collected information on all-drugs intake and for sub-categories. The protocol included a comprehensive list of drugs that patients could be taking, such as analgesics or non-steroidal anti-inflammatory drugs (NSAIDs), triptans, opioids ergot-derivates and other acute drugs (although we presented only data on NSAIDs and triptans, since they are the most commonly used drugs). Patients were asked to refer the number of intakes per month and, if they could not recall it, they could provide it by ranges, namely 1-5, 5-10, 10-15, 15-20, 20-25, 25-30, 30-40, 40-50, 50-60, 60-70, 70-80, 80-90 or 90+ per month. In this case, the central value was taken for each category with a range, while the number 90 was assumed for the last category.

Based on the same protocol used in a study on medication overuse headache, three main cost categories were identified for this study [15]. All cost categories were referred to the three months prior to protocol completion: indirect costs, direct healthcare cost, and direct non-medical costs, whose sum represented the total cost incurred in the previous trimester.

Indirect costs are based upon LPT. Patients were asked to refer their net salary, either giving the precise amount or relying on pre-defined categories (<500 €; 500€–750€; 750€–1000€; 1000€–1500€; 1500€–2000€; 2000€–4000€; 4000€–7000€; 7000€–10000€; >10000€): in the latter case, we used the median value, and the highest and lowest categories were taken as absolute values (i.e. 500€ and 10000€). We then determined the gross yearly salary and divided it by 230 standard working days to get the gross daily salary, which was them multiplied for the day-equivalent LPT to determine the three-month indirect cost.

Direct healthcare cost, which included those incurred for medications, medical consultations, diagnostic procedures and for other treatments connected to headache. In addition to the quantification of the amount of drugs for the acute headache treatment, patients were also asked to report the use of prophylactic compounds, non-pharmacological treatments, such as nutraceuticals, behavioural and physical therapies, diagnostics and access to health services (i.e. neurological examinations or access to emergency room). These medications and treatment can be either partially subsidized by the Italian National Health System (NHS) or not subsidized at all, depending on pharmaceutical classes and treatment kind, and thus we considered the total price for each drug, regardless of their eventual subsidy. We relied on standard prices currently found on the market in 2015, as identified from a private site for health care professionals [16]. The list of drugs was kept at the same price for the whole period: the only amendment made in course was the inclusion of the three monoclonal antibodies (Erenumab, Galcanezumab and Fremanezumab) which were marketed during the study period.

Finally, direct non-medical costs, included costs of informal care for housework and for baby-sitting, were directly referred by patients specifying the average cost.

**References**

1. Martin BC, Pathak DS, Sharfman MI, Adelman JU, Taylor F, Kwong WJ, Jhingran P. Validity and reliability of the migraine-specific quality of life questionnaire (MSQ Version 2.1). Headache. 2000;40(3):204-15. doi: 10.1046/j.1526-4610.2000.00030.x.
2. Raggi A, Giovannetti AM, Schiavolin S, Leonardi M, Bussone G, Grazzi L, Usai S, Curone M, Di Fiore P, D'Amico D. Validating the Migraine-Specific Quality of Life Questionnaire v2.1 (MSQ) in Italian inpatients with chronic migraine with a history of medication overuse. Qual Life Res. 2014;23(4):1273-7. doi: 10.1007/s11136-013-0556-9.
3. Stewart WF, Lipton RB, Simon D, Von Korff M, Liberman J. Reliability of an illness severity measure for headache in a population sample of migraine sufferers. Cephalalgia. 1998;18(1):44-51. doi: 10.1046/j.1468-2982.1998.1801044.x.
4. D'Amico D, Mosconi P, Genco S, Usai S, Prudenzano AM, Grazzi L, Leone M, Puca FM, Bussone G. The Migraine Disability Assessment (MIDAS) questionnaire: translation and reliability of the Italian version. Cephalalgia. 2001;21(10):947-52. doi: 10.1046/j.0333-1024.2001.00277.x.
5. Ustün TB, Chatterji S, Kostanjsek N, Rehm J, Kennedy C, Epping-Jordan J, Saxena S, von Korff M, Pull C; WHO/NIH Joint Project. Developing the World Health Organization Disability Assessment Schedule 2.0. Bull World Health Organ. 2010;88(11):815-23. doi: 10.2471/BLT.09.067231.
6. Garin O, Ayuso-Mateos JL, Almansa J, Nieto M, Chatterji S, Vilagut G, Alonso J, Cieza A, Svetskova O, Burger H, Racca V, Francescutti C, Vieta E, Kostanjsek N, Raggi A, Leonardi M, Ferrer M; MHADIE consortium. Validation of the "World Health Organization Disability Assessment Schedule, WHODAS-2" in patients with chronic diseases. Health Qual Life Outcomes. 2010;8:51. doi: 10.1186/1477-7525-8-51.
7. Kosinski M, Bayliss MS, Bjorner JB, Ware JE Jr, Garber WH, Batenhorst A, Cady R, Dahlöf CG, Dowson A, Tepper S. A six-item short-form survey for measuring headache impact: the HIT-6. Qual Life Res. 2003;12(8):963-74. doi: 10.1023/a:1026119331193.
8. Beck AT, Steer RA, Brown GK (1996). Beck depression inventory manual (2nd ed.). The Psychological Corporation, Harcourt, Brace & Company, San Antonio (TX).
9. Ghisi M, Flebus GB, Montano A, Sanavio E, Sica C (2006). Beck Depression Inventory-II. Manuale Italiano. Organizzazioni Speciali, Firenze
10. Spielberger CD (1989) State-Trait Anxiety Inventory: Bibliography, 2nd edn. Consulting Psychologists Press, Palo Alto (CA)
11. Predabissi L, Santinello M(1989). Inventario per l’ansia di stato e di tratto Nuova versione italiana dello S.T.A.I. – Forma Y. Organizzazioni Speciali, Firenze
12. Lipton RB, Bigal ME, Ashina S, Burstein R, Silberstein S, Reed ML, Serrano D, Stewart WF; American Migraine Prevalence Prevention Advisory Group. Cutaneous allodynia in the migraine population. Ann Neurol. 2008;63(2):148-58. doi: 10.1002/ana.21211.
13. Brown KW, Ryan RM. The benefits of being present: mindfulness and its role in psychological well-being. J Pers Soc Psychol. 2003;84(4):822-48. doi: 10.1037/0022-3514.84.4.822.
14. Raggi A, Covelli V, Guastafierro E, Leonardi M, Scaratti C, Grazzi L, Bartolini M, Viticchi G, Cevoli S, Pierangeli G, Tedeschi G, Russo A, Barbanti P, Aurilia C, Lovati C, Giani L, Frediani F, Di Fiore P, Bono F, Rapisarda L, D'Amico D. Validation of a self-reported instrument to assess work-related difficulties in patients with migraine: the HEADWORK questionnaire. J Headache Pain. 2018;19(1):85. doi: 10.1186/s10194-018-0914-7.
15. Raggi A, Leonardi M, Sansone E, Curone M, Grazzi L, D'Amico D. The cost and the value of treatment of medication overuse headache in Italy: a longitudinal study based on patient-derived data. Eur J Neurol. 2020;27(1):62-e1. doi: 10.1111/ene.14034.
16. Federfarma. <https://www.federfarma.it/Farmaci-e-farmacie/Cerca-un-farmaco.aspx>
