# Supplementary material for: Efficacy of mindfulness added to treatment as usual in patients with chronic migraine and medication overuse headache: a phase-III single-blind randomized-controlled trial (the MIND-CM study)
Source: J Headache Pain. 2023 Jul 14;24(1):86. doi: 10.1186/s10194-023-01630-0 (PMC10347788; doi:10.1186/s10194-023-01630-0)
Supplement: Supplementary file 2 — Additional file 2. [file 10194_2023_1630_MOESM2_ESM.docx]

**Additional File 2**

Online-only Supplementary Material to “Efficacy of Mindfulness added to treatment as usual in patients with Chronic Migraine and Medication Overuse Headache: A Phase-III Single-Blind randomized-controlled trial (the MIND-CM study)”

**Authors**: Licia Grazzi, Domenico D’Amico, Erika Guastafierro, Greta Demichelis, Alessandra Erbetta, Davide Fedeli, Anna Nigri, Emilio Ciusani, Barbara Corso, Alberto Raggi.

**Journal:** Journal of Headache and Pain

**SUPPLEMENTARY RESULTS**

**Supplementary Figure 1.** Migraine-Specific Quality of Life (MSQ) time by group analysis from baseline to month 12.


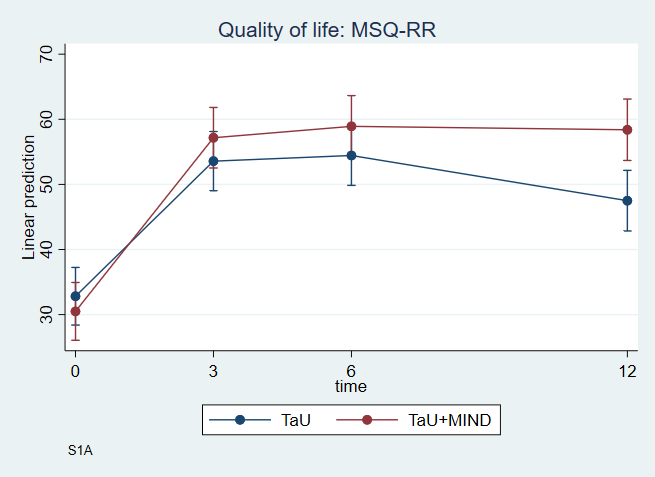


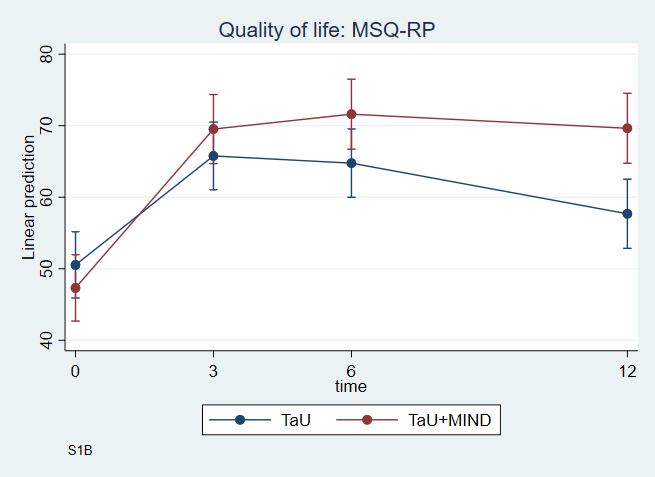


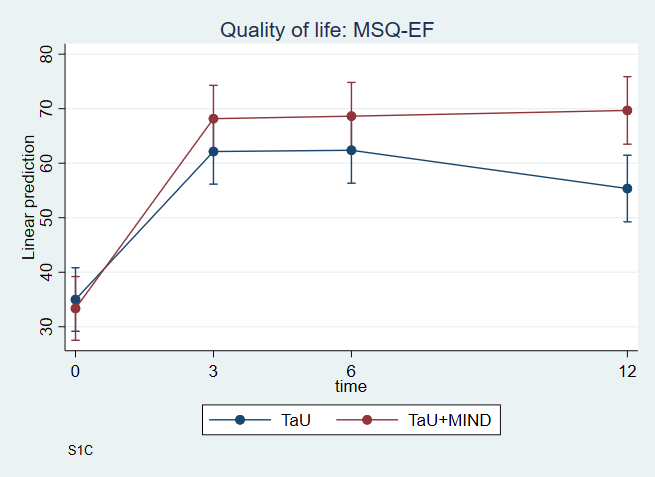


*Notes.* Figure S1A, MSQ-Role Restriction; Figure S1B, MSQ-Role Prevention; Figure S1C, MSQ-Emotional Function

**Supplementary Figure 2.** Migraine Disability Assessment (MIDAS) time by group analysis from baseline to month 12.


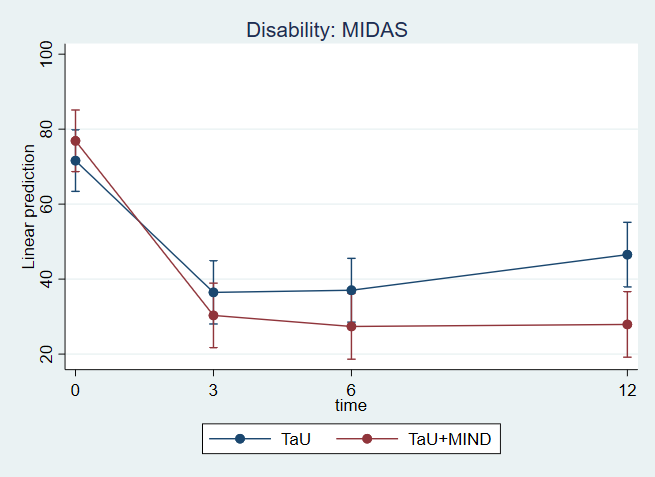


**Supplementary Figure 3.** 12-item WHO Disability Assessment Schedule (WHODAS-12) time by group analysis from baseline to month 12.


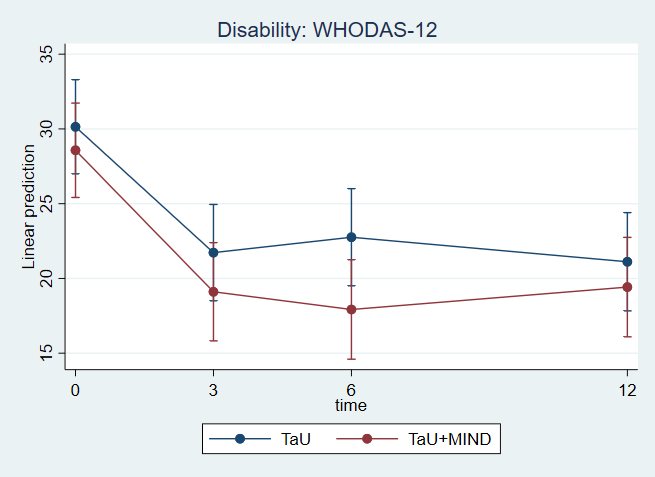


**Supplementary Figure 4.** 6-item Headache Impact Test (HIT-6) time by group analysis from baseline to month 12.


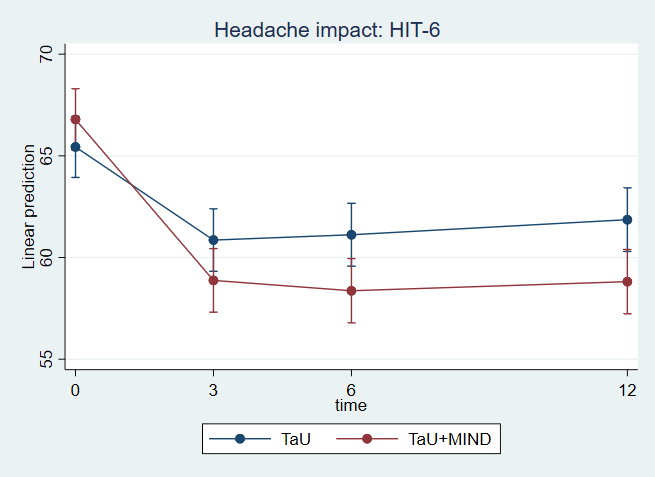


**Supplementary Figure 5.** Beck Depression Inventory, 2^nd^ version (BDI-II) time by group analysis from baseline to month 12.


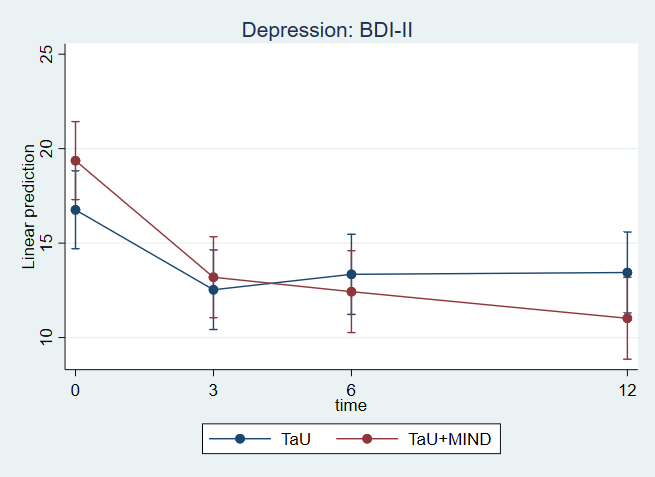


**Supplementary Figure 6.** State-Trait Anxiety Inventory Y-form (STAI-Y) time by group analysis from baseline to month 12.


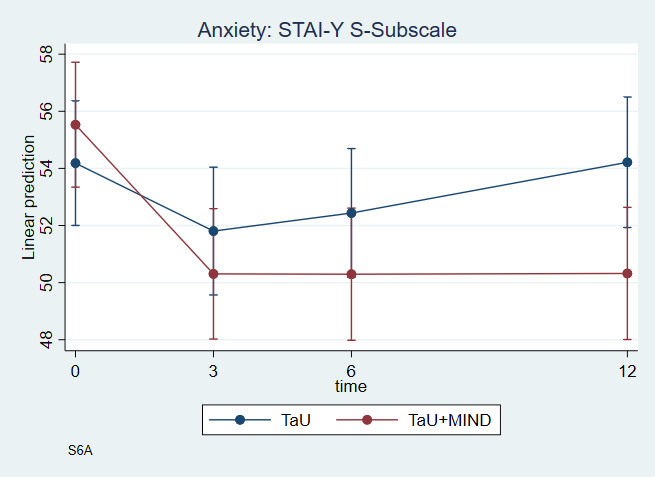


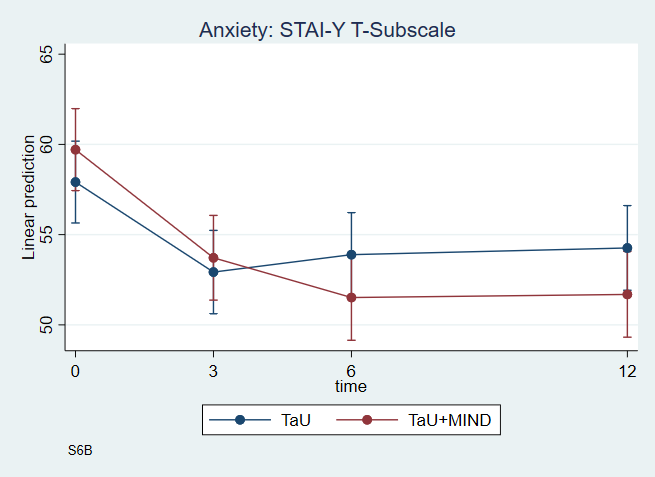


*Notes.* Figure S6A, STAI-Y State subscale; Figure S6B, STAI-T Trait subscale

**Supplementary Figure 7.** 12-items Allodynia Symptoms Checklist (ASC-12) time by group analysis from baseline to month 12


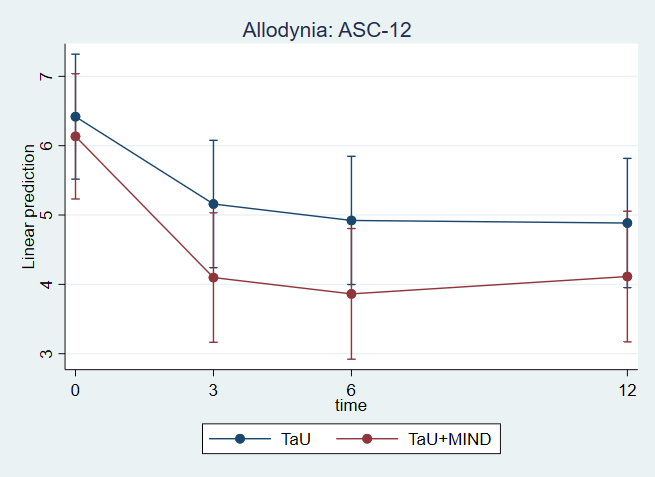


**Supplementary Figure 8.** Mindful Attention and Awareness Scale (MAAS) time by group analysis from baseline to month 12


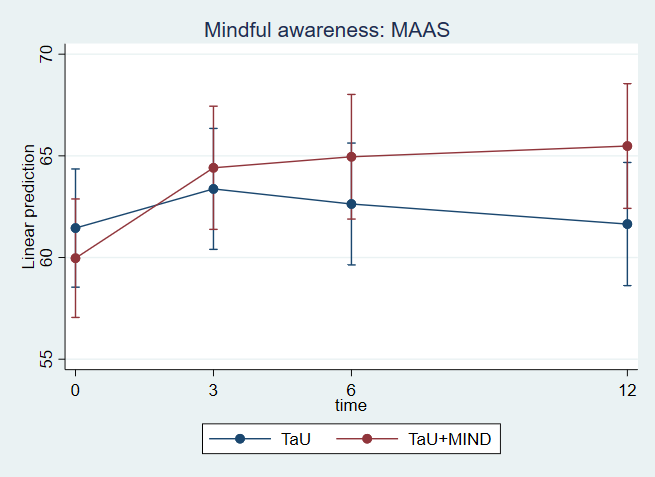


**Supplementary Figure 9.** HEADWORK questionnaire time by group analysis from baseline to month 12


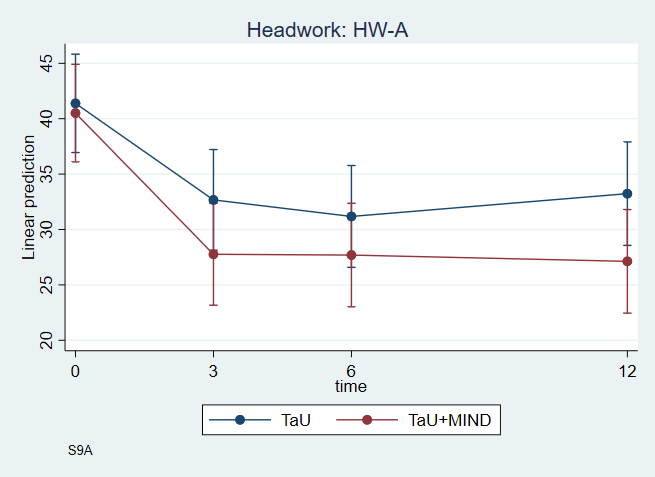


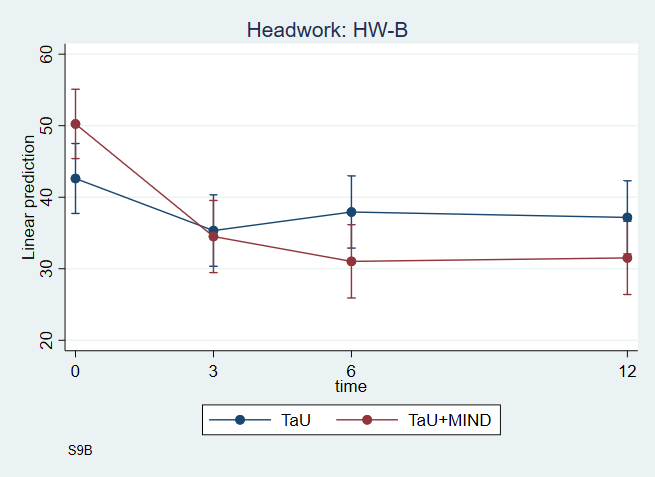


*Notes.* Figure S9A, HEADWORK-A subscale (Work-related difficulties); Figure S9B, HEADWORK-B subscale (Factors contributing to work difficulties).

**Supplementary Figure 10.** Total cost and breakdown of the three cost subcategories, time by group analysis from baseline to month 12.


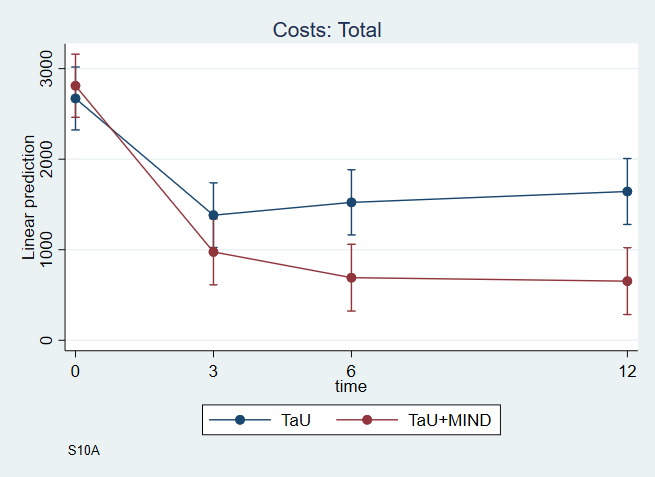


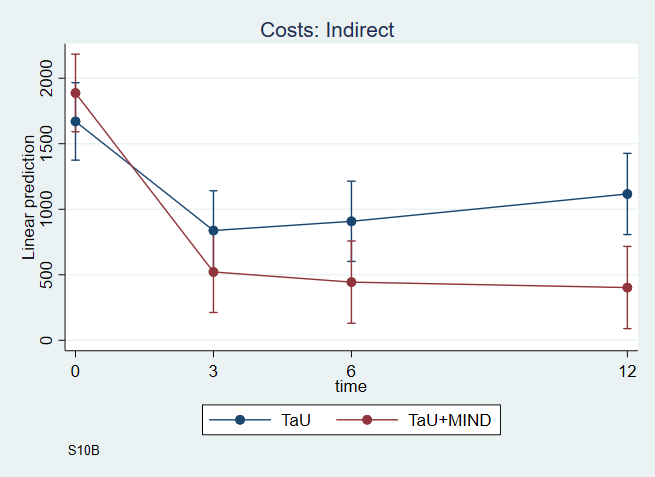


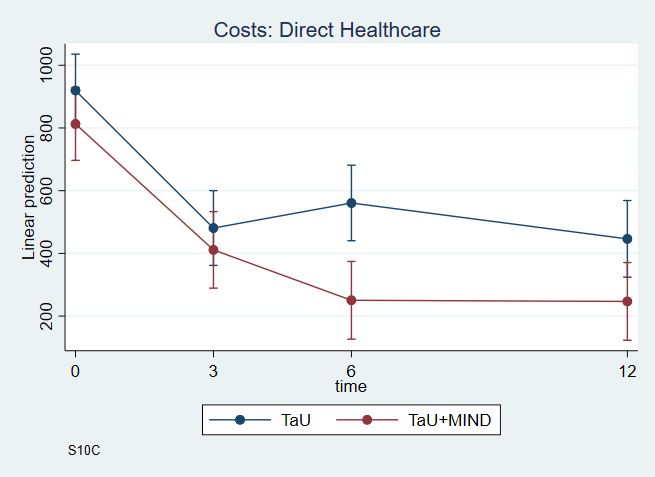


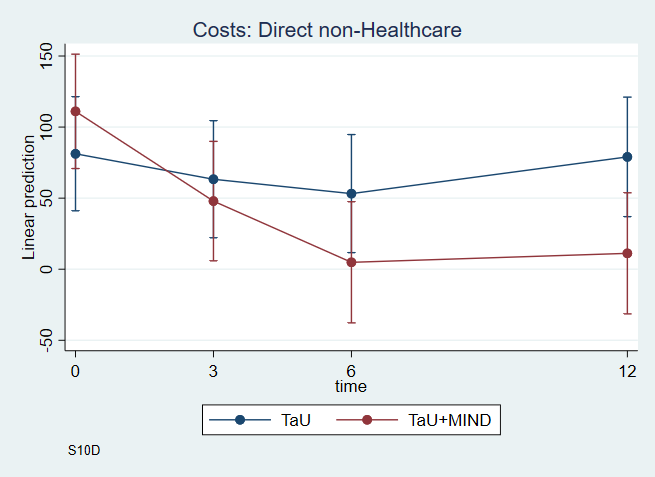


*Notes.* Figure S10A, total cost; Figure S10B, indirect cost; Figure S10C, direct healthcare cost; Figure S10D, direct non-medical cost;

**Supplementary Figure 11.** Time by group analysis for NSAIDs intake and reduction from baseline to month 12.


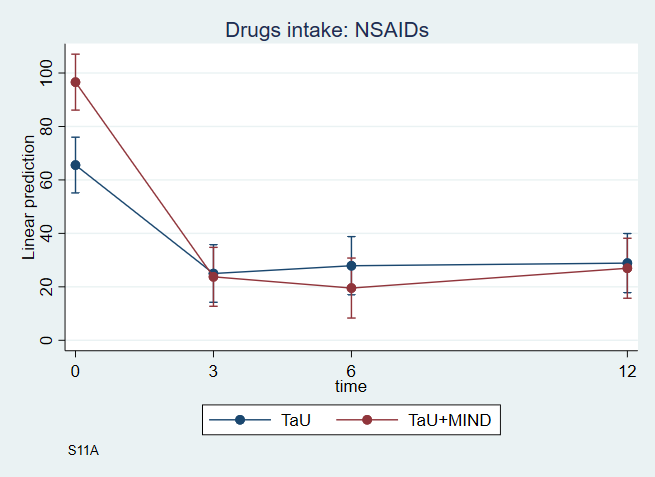


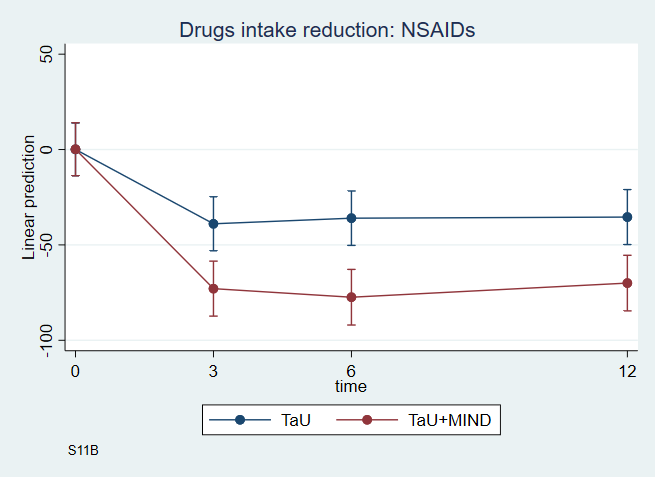


*Notes.* Figure S11A, NSAIDs intake between baseline and month 12; Figure S11B, reduction in NSAIDs intake from baseline to month 12

**Supplementary Figure 12.** Time by group analysis for triptans intake and reduction from baseline to month 12.


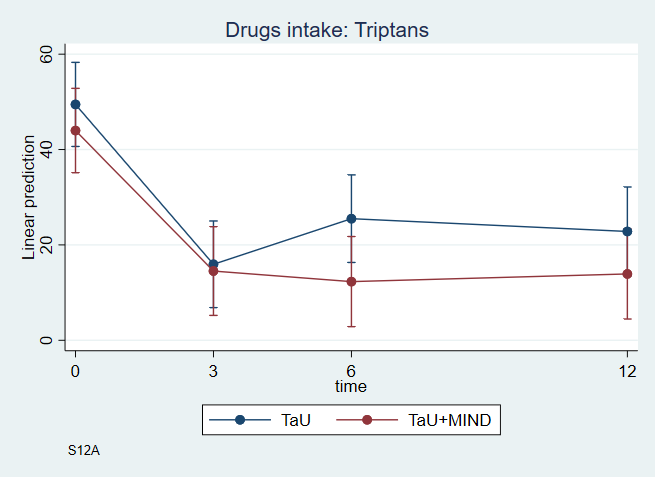


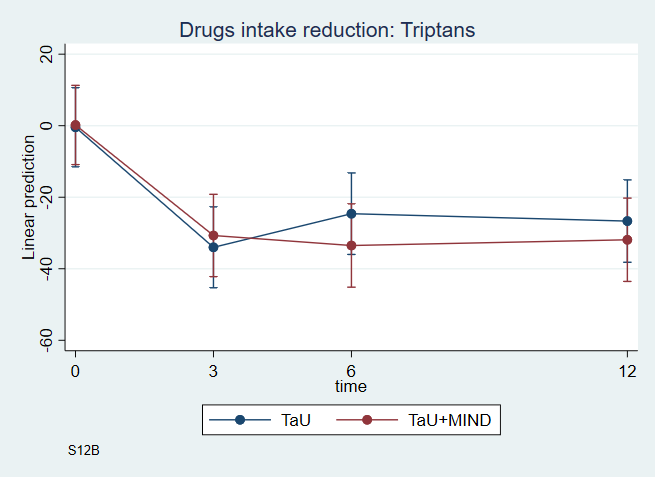


*Notes.* Figure S12A, triptans intake between baseline and month 12; Figure S12B, decrease in triptans intake from baseline to month 12

**Supplementary Table 1**. Achievement of HIT-6 score reduction ≥6 points by group

| Time point | 3-Month follow-up (N=162) | | 6-Month follow-up (N=157) | | 12-Month follow-up (N=154) | |
| --- | --- | --- | --- | --- | --- | --- |
| Group allocation | TaU Group | TaU+MIND Group | TaU Group | TaU+MIND Group | TaU Group | TaU+MIND Group |
| HIT-6 reduction ≥6 from baseline | 36/83  (43.4%) | 50/79  (63.3%) | 33/81  (40.7%) | 47/76  (61.8%) | 29/78  (37.2%) | 45/76  (59.2%) |
| Chi-Squared test (p-value) | 6.4  (p=0.011) | | 7.0  (p=0.008) | | 7.5  (p=0.006) | |
| OR (95% CI) | 2.25  (1.20-4.23) | | 2.36  (1.24-4.47) | | 2.45  (1.28-4.69) | |

*Note.* TaU, treatment as usual; TaU+MIND, treatment as usual with six mindfulness-based sessions; HIT-6, six-item Headache Impact Test; OR, odds ratio; 95% CI, 95% confidence interval
